# Current Methods in the Molecular Typing of *Mycobacterium tuberculosis* and Other Mycobacteria

**DOI:** 10.1155/2014/645802

**Published:** 2014-01-05

**Authors:** Tomasz Jagielski, Jakko van Ingen, Nalin Rastogi, Jarosław Dziadek, Paweł K. Mazur, Jacek Bielecki

**Affiliations:** ^1^Department of Applied Microbiology, Institute of Microbiology, Faculty of Biology, University of Warsaw, Ilii Miecznikowa 1, 02-096 Warsaw, Poland; ^2^Department of Medical Microbiology, Radboud University Nijmegen Medical Centre, P.O. Box 9101, 6500 HB Nijmegen, The Netherlands; ^3^WHO Supranational TB Reference Laboratory, TB and Mycobacteria Unit, Institut Pasteur de Guadeloupe, BP 484, 97183 Abymes Cedex, France; ^4^Mycobacterium Genetics and Physiology Unit, Institute of Medical Biology, Polish Academy of Sciences, Lodowa 106, 93-232 Łódź, Poland; ^5^Department of Genetics, Stanford University Medical School, Stanford, CA 94305-5120, USA

## Abstract

In the epidemiology of tuberculosis (TB) and nontuberculous mycobacterial (NTM) diseases, as in all infectious diseases, the key issue is to define the source of infection and to disclose its routes of transmission and dissemination in the environment. For this to be accomplished, the ability of discerning and tracking individual *Mycobacterium* strains is of critical importance. Molecular typing methods have greatly improved our understanding of the biology of mycobacteria and provide powerful tools to combat the diseases caused by these pathogens. The utility of various typing methods depends on the *Mycobacterium* species under investigation as well as on the research question. For tuberculosis, different methods have different roles in phylogenetic analyses and person-to-person transmission studies. In NTM diseases, most investigations involve the search for environmental sources or phylogenetic relationships. Here, too, the type of setting determines which methodology is most suitable. Within this review, we summarize currently available molecular methods for strain typing of *M. tuberculosis* and some NTM species, most commonly associated with human disease. For the various methods, technical practicalities as well as discriminatory power and accomplishments are reviewed.

## 1. Introduction

The genus *Mycobacterium* contains more than 140 species [1], which are separated in three major groups, that is, *M. tuberculosis* complex (MTBC), *M. leprae*, and mycobacteria other than MTBC and *M. leprae*, collectively referred to as nontuberculous mycobacteria (NTM). *Mycobacterium tuberculosis*, the most prominent member of the MTBC, is an obligate human pathogen and the causative agent of tuberculosis (TB), which remains one of the leading global public health problems. According to the World Health Organization (WHO), over 9 million new cases of TB occur each year, resulting in approximately 2 million deaths worldwide [2]. Conversely to *M. tuberculosis*, for which no environmental reservoir exists, NTM are ubiquitous organisms and are readily isolated from environmental sources, including soil and both natural and artificial water systems [3]. Despite reportedly low virulence of NTMs for immunocompetent human hosts, an increase in their isolation frequencies has been seen in the last decade, particularly in countries where TB incidence is on decrease [4, 5].

In the epidemiology of TB and other mycobacterioses, as in all infectious diseases, the key issue is to define the source of infection and to disclose its routes of transmission and dissemination in the environment. For this to be accomplished, the ability of discerning and tracking individual *Mycobacterium *strains is of critical importance. The earliest discriminatory methods relied upon phenotypic characteristics, such as colony morphology, susceptibility to antituberculosis drugs, or mycobacterial phage typing. The usefulness of these methods is seriously limited by the phenotypic variability of mycobacteria. For instance, drug susceptibility patterns may change for the same isolate as it acquires resistance to specific drugs in the course of treatment. Otherwise, the limiting factor of phage typing is the low number of recognized mycobacteriophages [6–8]. Discrimination between the strains based on their biochemical and serological characteristics is even more unsuccessful [9, 10]. A turning point in this quest was the development of molecular biology tools in the mid 1980s. The DNA-based techniques have revolutionized the epidemiology of TB and other mycobacterial diseases, since they allow querying the whole genome that is unique and relatively stable for each strain.

In terms of genetic heterogeneity, the MTBC and NTM are vastly different and this has strong implications for the choice of typing methods and the achievable levels of discrimination.

*Mycobacterium tuberculosis* complex constitutes a remarkably genetically homogeneous group. This is perhaps best illustrated by the fact that, within the ribosomal DNA (rDNA) operon, not only genes encoding various types of rRNA but also regions between those genes, such as internal transcribed spacer (ITS) regions, which in many bacteria and fungi are highly polymorphic and thus are useful for identification and typing to subspecies or strain level, show complete conservation among members of the *M. tuberculosis* complex [11]. Likewise, many structural genes of *M. tuberculosis* complex show important sequence conservation, with an estimated rate of synonymous mutations of 0.01–0.03% [12–14]. Additionally, the lack of significant evidence for horizontal gene transfer between *M. tuberculosis* genomes speaks in favor of clonal evolution in *M. tuberculosis* complex [12–15]. This in turn renders strain-level discrimination of *M. tuberculosis* by means of molecular typing approaches challenging. However, recent studies have shown that the genetic diversity of the MTBC is much higher than previously assumed and that this genomic variance is attributed to the single nucleotide polymorphisms (SNPs), with potential impact on pathobiological phenotype [16, 17].

As for the NTM, the evolutionary time of divergence is believed to be much larger than that for the *M. tuberculosis* complex and this implies that less than a whole genome can be queried to obtain a highly discriminatory typing result [18].

Within this review, we summarize currently available molecular methods for strain typing of mycobacteria. For the various techniques, technical practicalities as well as discriminatory power and accomplishments are reviewed.

## 2. Restriction Site Analysis of Genomic DNA

First attempts of molecular typing of *M. tuberculosis* were based on restriction enzyme analysis of bacterial DNA (REA). In principle, chromosomal DNA of the analyzed strains is digested using various restriction enzymes and the resulting fragments are separated and visualized by gel electrophoresis. The obtained pattern of DNA fragments (*genetic fingerprint*) is characteristic for each strain. However, using the original procedure, the sensitivity of the method is rather limited due to technical difficulties in providing a high-resolution electrophoretic separation of fragments within a broad range of sizes. Also, when more restriction enzymes are used, the high number of DNA fragments makes a reliable analysis impossible [19]. Therefore, new methods have been proposed for a more accurate separation of DNA molecules, such as REA-PFGE (pulsed-field gel electrophoresis), which guarantees high resolution of the restriction patterns. More commonly used methods, which are modifications of the traditional REA, employ DNA hybridization assay in the process of specific pattern detection. Specifically, after electrophoresis, the separated DNA fragments are denatured in situ and transferred onto a membrane, which is then incubated with a radiolabeled probe (Southern blot) for the target sequence. Hybridization signals are visualized on autoradiography. The Southern blot technique was first applied to the analysis of restriction fragment length polymorphism (RFLP), which is hence a combination of REA and hybridization technology. Early studies utilizing RFLP methodology indicated that strains of *M. tuberculosis* display a considerably low degree of genetic diversity. However, such interpretation of the results was actually misleading since probes used in those studies targeted highly conserved regions and were of low specificity [20–22]. The resolution of the RFLP method increased substantially when insertion sequences (IS) were identified and used in probe construction [23, 24]. Different repetitive sequences found in the genomes of *M. tuberculosis* and NTM are an important source of genetic polymorphism and provide reliable markers allowing the determination of genetic relationships at both species and strain levels.

## 3. Pulsed-Field Gel Electrophoresis (PFGE)

A method related to REA, pulsed-field gel electrophoresis (PFGE), enables the separation of large DNA fragments, up to 10 Mb. In contrast, by using conventional electrophoresis, the threshold length exists at about 50 kb. The principle of the PFGE system is based on the application of an electric field that periodically changes its orientation across a gel matrix. This is achieved by varying the duration of the electrical pulse and shifting the direction of the current frequently. In general, the PFGE procedure involves digestion of chromosomal DNA with rare cutting restriction endonucleases, followed by agarose gel electrophoresis and analysis of the resolved electrophoretic patterns. A crucial step is the preparation of genomic DNA. Since large DNA molecules are prone to shearing and crushing, DNA is isolated in a gentle manner by first embedding a suspension of the organism in agarose plugs, lysing the cells in situ, and digesting the chromosomal DNA with restriction enzymes. The plugs are then loaded into the gel wells and sealed into place with agarose. After the electrophoresis, the resulting banding patterns are compared, using a predefined set of criteria for strain relatedness [25]. Although the PFGE patterns are well reproducible and the overall discriminatory power of the method is high, a number of limitations are apparent. Firstly, the method is technically demanding and cost intensive. Secondly, it requires intact DNA for restriction enzyme treatment. Thirdly, the PFGE method has a long turn-over time, as the whole protocol usually takes not less than a week. Finally, no standardized procedure for performing PFGE has yet been recommended. Despite these disadvantages, the PFGE typing was successfully used to differentiate between strains of *M. tuberculosis* [26], *M. bovis* [27], and *M. bovis* BCG [28]. However, the PFGE typing is rarely used in *M. tuberculosis* complex due to technical, time, and cost considerations, as mentioned above. Moreover, PFGE analysis does not always generate sufficient discrimination between the strains [26, 29, 30].

Quite oppositely, PFGE often remains the most powerful typing system for nontuberculous mycobacteria. The method has been applied, with different degrees of success, to both slow-growing NTM species, including *M. kansasii* [31, 32], *M. avium*-*M. intracellulare* complex [33], *M. gordonae* [34], and *M. haemophilum* [35], and rapidly growing mycobacteria, such as *M. fortuitum* [36], *M. chelonae* [37], and *M. abscessus* [37, 38].

## 4. IS*6110*-RFLP Analysis

Study of the complete *M. tuberculosis* H_37_Rv reference strain genome sequence revealed a relatively large amount of repetitive DNA elements [39]. Those elements vary in length, structure, and localization. Two main groups can be distinguished, that is, tandem repeats (TR) and interspersed repeats (IR). The first are short monomeric sequences (up to 100 bp) organized as head-to-tail arrays, whereas the latter are scattered as individual copies throughout the entire genome. An important class of IR sequences is insertion sequences (IS), which are mobile genetic elements.

The best known and investigated insertion sequence is IS*6110* first recognized by Thierry et al. in the early 1990s [40–43]. The IS*6110* sequence belongs to the IS*3* family and is a 1,355 bp long with unique 28 bp terminal inverted repeats (TIR). The region between those repeats includes two overlapping reading frames, *orfA* and *orfB*, encoding for a transposase, an enzyme responsible for transposition of the insertion sequence. The IS*6110* is found within the *M. tuberculosis* complex, and, in most members of the complex, the sequence is present at multiple copies, although *M. bovis* normally contains only one copy. In general, the copy number of IS*6110* ranges from 0 to 25 and depends on the frequency of transposition, which is largely conditioned by the nature of the genomic region at which transposition occurs [44]. Although the IS*6110* can be integrated into any place on the chromosome, there are regions with higher frequency of transposition. The so-called integration hot spots are usually located within coding regions of *M. tuberculosis* DNA [44, 45].

Differences in the copy number and locations within the genome, responsible for the high degree of IS*6110* polymorphism, have predisposed this sequence to be used as a specific molecular marker for genotyping of *M. tuberculosis* strains [46, 47].

**Figure 1:**
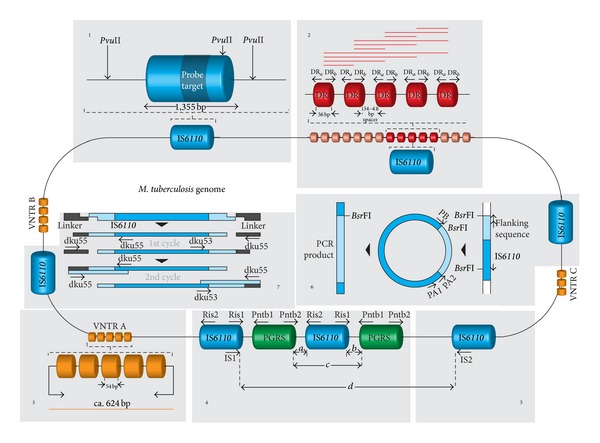
Schematic representation of the chromosome of a hypothetical *Mycobacterium tuberculosis* complex isolate with marked repetitive elements as targets for different typing methods. The principle of those methods is pictorially outlined. (1) In IS*6110*-RFLP typing, mycobacterial DNA is cleaved with the restriction endonuclease *Pvu*II, and the resulting fragments are separated electrophoretically on an agarose gel, transferred onto a nylon membrane by Southern blotting, and hybridized to a probe complementary to the 3′-end of the IS*6110* (probe target) yielding a characteristic banding pattern, in which every band represents a single IS*6110* element. (2) Spoligotyping relies upon PCR amplification of a single direct repeat (DR) locus which harbours 36 bp direct repeats interspersed with unique 34–41 bp spacer sequences. The PCR products (red horizontal lines) are hybridized to a membrane containing 43 oligonucleotides corresponding to the spacers from *M. tuberculosis* H_37_Rv and *Mycobacterium bovis* BCG. The presence or absence of each of those 43 spacers in the DR region of the analysed isolate will be represented as the pattern of positive or negative hybridization signals. (3) The variable numbers of tandem repeat loci (VNTR) or mycobacterial interspersed repetitive units (MIRU) are PCR-amplified and the obtained products (yellow horizontal line) are sized on agarose gels to deduce the number of repeats in each individual locus. (4, 5) Two PCR-based typing methods, that is, double-repetitive-element PCR (DRE-PCR) and amplityping, are designed to amplify DNA between clusters of IS*6110* and polymorphic GC-rich sequences (PGRS) or between clusters of IS*6110 *elements, respectively. Different distances between the repetitive elements and their different copy numbers result in variability of banding patterns, composed of DNA fragments amplified (*a*–*d*) and produced for individual isolates. (6) A heminested inverse PCR (HIP) depends on the amplification of the 5′-end of the IS*6110* sequence along with its upstream flanking sequence, bordered by the closest *Bsr*FI site. The size and number of PCR amplicons generated depend on the number of copies of IS*6110*. (7) Ligation-mediated PCR (LM-PCR) procedure allows, by introducing specifically designed linkers, amplifying the flanking sequences on both sides of the IS*6110* element. Names and positions of the PCR primers were excerpted from the original papers. For more details, read the text.

IS*6110*-based typing is still among the most widely applied genotyping methods in molecular epidemiological studies of *M. tuberculosis*. The method includes digestion of genomic DNA with *Pvu*II restriction enzyme that cleaves the IS*6110* sequence only once, generating DNA fragments that are separated through gel electrophoresis, then transferred onto a membrane, and hybridized with a peroxidase-labelled probe complementary to part of the 3′-end of the IS*6110* sequence. As a result, every visualized fragment represents a single copy of IS*6110* surrounded by different in length flanking DNA (Figure 1(1)) [48]. Since the IS*6110*-RFLP methodology has been standardized and published, recommendations have been adopted by most of the research groups; the fingerprints generated in different laboratories can be compared and catalogued [49, 50]. The method is highly discriminatory and reproducible. An important characteristic of IS*6110*-RFLP typing is the stability of its profiles over time, allowing distinguishing epidemiologically related from unrelated isolates. Specifically, the half-time of change in IS*6110*-RFLP pattern was estimated to be *ca*. 3-4 years [51, 52]. Stability of the IS*6110*-RFLP patterns depends on the transposition process frequency; the more common is the transposition, the less stable is the number of the IS*6110* element in the genome. However, several important limitations exist for the IS*6110*-RFLP method. Firstly, there is a need for large amounts of high quality DNA (2 *μ*g) for restriction enzyme digestion and therefore requires time consuming (up to several weeks) bacterial culturing. Secondly, the method is technically demanding and requires sophisticated and expensive computer software as well as experienced personnel of high technical expertise. Finally, the discriminatory power of IS*6110*-RFLP typing is insufficient for those strains whose copy number of IS*6110* is 6 or less (the so-called low-copy strains are seen among *M. bovis* isolates from cattle or *M. tuberculosis* isolates from Asia) [53–56]. In addition, some NTM have multiple copies of sequences that are homologous to IS*6110* and may thus hybridize with the IS*6110* probe [57].

Despite these limitations, the IS*6110*-RFLP method remains one of the most commonly used approaches for *M. tuberculosis* typing and was long considered the gold standard technique in the molecular epidemiological investigations of TB.

## 5. IS*6110*-Based PCR Fingerprinting

The IS*6110* is a target sequence in many methods currently used for molecular typing of *M. tuberculosis*. Among these, the most important is the mixed-linker PCR (ML-PCR) [58], ligation-mediated PCR (LM-PCR) [60], and fast ligation-mediated PCR (FliP) [59]. All these methods follow a similar four-step algorithm including genomic DNA fragmentation using restriction enzymes that generate protruding ends (i), ligation of those fragments with synthetic oligonucleotide linkers or adaptors (ii), amplification of the ligation products with one primer specific for the IS*6110* and a second primer complementary to a linker (iii), and analysis of the amplicons (iv).

In ML-PCR and FliP methods the products are 3′ fragments of IS*6110*, whereas in LM-PCR the IS*6110*-flanking sequence on the 5′ side is amplified. (In the original LM-PCR procedure flanking sequences on both sides of the IS*6110* are amplified by using primers that are complementary to both termini of the IS*6110* sequence and one of the primers is also complementary to the shorter strand of the linker (Figure 1(7)) [68].)

All those methods are highly reliable and exhibit significant discriminatory potential, albeit slightly lower than that of IS*6110*-RFLP analysis [59, 69–71].

This potential can even be increased when using heminested inverse PCR (HIP). This method relies on amplification of a 5′ part of the IS*6110* and its flanking sequence up to the proximal *Bsr*FI restriction site [61]. Briefly, chromosomal DNA is cut with the restriction endonuclease *Bsr*FI and the restriction fragments are then self-ligated at low DNA concentration. The resulting circular molecules comprised of the 5′-end of the IS*6110* and its flanking region are subjected to PCR using primers that anneal to the IS at sites between its 5′-end and the closest *Bsr*FI site. Products of amplification vary in length depending on the length of the flanking sequence and are visualized by agarose gel electrophoresis (Figure 1(6)). The HIP method is highly reproducible and its discriminatory power is equivalent to that of standard IS*6110*-RFLP analysis but is much simpler and faster in performance [61, 72].

Another PCR-based IS*6110* typing method is amplityping. Here, outward-oriented primers hybridize with the ends of the IS*6110* sequence, so that DNA separating adjacent copies of this element on the genome is amplified (Figure 1(5)) [73–75]. A variant of the aforesaid method uses only a single primer, targeted to the terminal inverted repeat sequences of the IS*6110* [76, 77]. Differences in the length of the amplicons reflect the distance between the IS elements and are analysed by standard gel electrophoresis. An important limitation of this typing approach is that production of the PCR amplicons is dependent on the priming sites within the ISs being close enough for efficient PCR.

All PCR-based typing assays, targeting the IS*6110*, are easy-to-perform, time saving and require relatively small amounts of genomic DNA, which makes them applicable to nonviable organisms or directly to clinical specimens, without culture. The resolution of the IS*6110*-*PCR analysis may not be sufficient enough to differentiate among the isolates; however* when supplemented with an additional restriction digestion step, the differentiation capacity of the method is comparable to IS*6110*-RFLP [78]. *Apart from some technical difficulties, such as nonspecific priming, still* the major drawback of the methods discussed is the lack of discriminatory power for typing of the isolates with low copy numbers of IS*6110* [74–77].

## 6. IS-Based Typing of NTM

Since a number of different ISs have been described in various NTM species, these genetic mobile elements have widely been applied for strain-level typing.

Two closely related insertion sequences IS*1245* and IS*1311* were found very useful to differentiate within the *M. avium* complex.

Based on the IS*1245* or IS*1311*-RFLP patterns, various clades of *M. avium* and its subspecies could be distinguished, related to infections in birds, pigs, and humans [79–81].

Other insertion elements that are known and have been used for identification rather than typing purposes of *M. avium* complex bacilli include IS*900* present in *M. avium* subsp. *paratuberculosis* [82], IS*901* present in *M. avium* subsp. *avium* [83], IS*902* present in *M. avium* subsp. *silvaticum* [84], and IS*666*, IS*1110*, and IS*1626*, whose distribution among *M. avium* isolates has been ill-studied [85–87]. The insertion sequences: IS*900*, IS*901*, IS*902*, and IS*1245,* can be used for the identification of various *M. avium* subspecies as well as for differentiation within those subspecies.

One of the most important observations made upon IS*1245* RFLP typing was that birds are infected by a genetically highly conserved type of *M. avium* strains invariably revealing the same three-band pattern, while the banding patterns of *M. avium* isolates of porcine and human origin revealed highly variable and multibanded patterns. Consequently, it was proposed to reserve the naming *M. avium*-*avium* for the bird-type isolates and to introduce the designation *M. avium hominissuis* for typical isolates from humans and pigs [81].

Beyond the *M. avium* complex, RFLP typing has been pursued only sporadically. Yet potentially useful insertion sequences have been described in a variety of species. These include IS*1407* in *M. celatum* [88], IS*1395* in *M. xenopi* [89], IS*1511*/*1512* in *M. gordonae* [90], IS*2404* in *M. ulcerans*, IS*2606* in *M. ulcerans* and *M. lentiflavum* [91], IS*1652* in *M. kansasii* [92], and IS*6120* in *M. smegmatis* [93].

## 7. Spoligotyping

Spoligotyping is currently one of the most frequently used PCR-based approaches for studying the phylogeography of *M. tuberculosis* complex. The spoligotyping method is based on the polymorphism at one particular genomic region, the so-called direct repeat (DR) locus, initially identified by Hermans et al. in the vaccine strain *M. bovis* BCG P3 [94]. The DR locus comprises a series of well-conserved 36 bp direct repeats (DRs) interspersed with unique, nonrepetitive spacer sequences of 34–41 bp. The DR and the adjacent variable sequence form a direct variant repeat (DVR). The DR locus belongs to the clustered regularly interspaced short palindromic repeats (CRISPRs) family of repetitive DNA. It is postulated that these elements are reminiscent of centromere-like structures with a possible role in replication partitioning [95]. In the spoligotyping method, the entire DR locus is amplified by PCR, using two inversely oriented primers complementary to the sequence of short DRs. The PCR products, of different sizes, are hybridized to a membrane with 43 covalently bound synthetic oligonucleotides representing the polymorphic spacers identified in *M. tuberculosis* H_37_Rv (spacers 1–19, 22–32, and 37–43) and *M. bovis* BCG (spacers 20-21 and 33–36). The hybridization signals are detected by chemiluminescence through biotin labeling of the PCR products (one of the primers is biotinylated) and a streptavidin-peroxidase conjugate system and then visualized by autoradiography (Figure 1(2)). Individual strains are differentiated by the number of the spacers that are missing from the complete 43-spacer set [62]. The lack of spacers is most probably the result of deletions mediated by various genetic mechanisms, such as homologous recombination or transposition (the DR region is a hot spot for IS*6110* integration) [96, 97].

Spoligotyping is a relatively simple, cost-effective, and high-throughput method, whose results are accurate and reproducible and are obtained in up to 2 days. The reliability of the results is linked to a high stability of the DR locus. The molecular clock of this genetic marker is believed to be very slow, since multiple *M. tuberculosis* isolates from the same patients corresponding to relapses or infections at different sites, even over time spans of several years, showed identical spoligotypes [98]. An important advantage of spoligotyping is its genuine sensitivity estimated at 10 fg of chromosomal DNA, equivalent to DNA from 2-3 bacterial cells [99], allowing the method to be applied directly in clinical samples, without the need for prior culture. Moreover, spoligotyping has proven to be useful for typing on nonviable cultures, Ziehl-Neelsen smear slides, or paraffin-embedded tissue sections [100, 101].

Given the binary (present/absent) format of the data, the spoligotyping results can easily be interpreted, computerized, and compared between different laboratories [102].

In 2006, an international spoligotyping database (SpolDB4) was released. The database, which is accessible online (http://www.pasteur-guadeloupe.fr:8081/SITVITDemo/), describes 1,939 STs (shared types, i.e., spoligotype patterns shared by two or more isolates) and 3,370 orphan types (i.e., spoligotype patterns reported for only single isolates) from a total of 39,295 *M. tuberculosis* complex isolates, from 122 countries, classified temporarily into 62 clades/lineages [103]. In a recently erected publicly available multimarker database named SITVIT, a total of 7105 spoligotype patterns (corresponding to 58,180 clinical isolates)—grouped into 2740 shared types containing 53,816 clinical isolates and 4364 orphan patterns—were incorporated (http://www.pasteur-guadeloupe.fr:8081/SITVIT_ONLINE/) [104].

Spoligotyping allows identification of *M. tuberculosis* complex isolates at the (sub)species level. For instance, the *M. tuberculosis* spoligotypes are characterized by the absence of spacers 33–36, whereas *M. bovis* spoligotypes usually lack spacers 39–43, and *M. bovis* BCG spoligotypes lack spacers 3, 9, and 16 [105]. Furthermore, spoligotyping allows identification of genotypes of significant, clinical, and epidemiological relevance. A clear example is the “Beijing” genotype, commonly encountered in the Beijing area, other regions of Asia, the former Soviet Union, and other geographical areas. Most of the “Beijing” genotype strains react only with the last 9 spacers (35–43) in the panel of 43 [105].

Spoligotyping has a lower level of discrimination than the IS*6110* RFLP typing, as evidenced in several studies [70, 106–110]. The introduction of 51 novel spacer sequences, mostly [45] from the DR region from the *M. canetti* genome only, slightly improved the resolution of the method [97]. The 68-spacer format, with 25 out of 51 new spacers, was shown to improve the discrimination for the *M. africanum* subspecies and for the East African-Indian (EAI) clade of *M. tuberculosis* [111, 112].

The reason for the limited discriminatory capacity of the spoligotyping method is due to the fact that it targets only a single genetic locus, covering less than 0.1% of the *M. tuberculosis* complex genome. Nevertheless, spoligotyping can be effectively used for the differentiation of *M. tuberculosis* strains with low IS*6110* copy numbers (≤5 bands in RFLP patterns) [54, 113]. As *M. tuberculosis* isolates with different spoligotypes invariably have distinct IS*6110* RFLP profiles, a genotyping strategy has been proposed, in which spoligotyping could be performed as a first-line, screening test, to be followed by another typing method of greater discriminatory power [108]. Spoligotyping, when used alone, is not sufficient for epidemiological linking studies. Furthermore, contaminated isolates and multistrain infections may not be detected by performing spoligotyping directly on clinical samples. However, this remark can be extended to any PCR-based technology, which is applied directly in clinical material.

Spoligotyping, when applied for nontuberculous mycobacteria, produced no signal, indicating the specificity of the method solely for *M. tuberculosis* complex [108].

Since the description of the spoligotyping method in its original form, another two formulations have been proposed. The first benefits from the Luminex technology, where the synthetic spacer oligonucleotide probes are immobilized on microspheres by means of covalent coupling, and detection is achieved via fluorochromes attached to the beads and hybridized PCR product. The Luminex platform, by eliminating the membrane step with the subjective manual data interpretation, provides, greater robustness and reproducibility. It is also well suited for high-throughput analysis, since it allows 96 isolates to be assayed simultaneously, as opposed to 45 isolates in a standard spoligotyping approach [112, 114].

A more recent alternative to a conventional spoligotyping scheme is a new multiplexed primer extension-based spoligotyping assay using automated matrix-assisted laser desorption ionization-time of flight mass spectrometry (MALDI-TOF MS). Spoligotyping by MALDI-TOF MS improves the classical reverse line blot hybridization assay with respect to reproducibility, throughput, process flow, ease of use, and data analysis [115]. An important limitation of these innovative, technologically refined spoligotyping assays is that they require advanced and expensive equipment which many laboratories, especially those in resource-constrained settings, cannot afford.

## 8. Methods Based on Minisatellite Sequences

*Mycobacterium tuberculosis* was among the first bacterial species in which tandem repeat loci resembling minisatellite loci in eukaryotic genomes were identified. The mycobacterial tandemly repeated sequences were, per analogiam to those in humans and animals, called variable number of tandem repeat (VNTR) loci. However, as new VNTR-type loci have been discovered, they have been referred to under different names.

The first described VNTRs were major polymorphic tandem repeat (MPTR) and exact tandem repeat (ETR), found in 5 (A–E) and 6 (A–F) loci, respectively [63]. The MPTR consists of a 10 bp repeated sequence separated by unique 5 bp spacer sequences. These repetitive DNA elements were identified in *M. tuberculosis* complex as well as in other mycobacteria, including* M. gordonae*, *M. kansasii*, or *M. szulgai* [116]. The MPTR has been shown particularly useful in typing of *M. kansasii*. RFLP analysis with MPTR as a probe has revealed the existence of multiple subtypes within this species [92]. These subtypes have later been shown to have different degrees of pathogenicity in humans [117]. Interestingly, the MPTR sequences are part of the 3′-end of genes belonging to the PPE proteins (named after the conserved Pro-Pro-Glu (PPE) motifs near the N terminus of the molecule). The polymorphism of the PPE proteins in their C-terminal domains, linked to the presence of the MPTR motifs, is speculated to be the source of antigen variability in *M. tuberculosis* [118].

The ETR elements, found exclusively in *M. tuberculosis* complex strains, contain repeats ranging in size from 53 to 79 bp. Sequencing of the ETRs revealed that all of the ETR loci were variable. Contrastingly, among MPTR loci, only one (MPTR-A) showed some polymorphism, upon sequencing analysis [63]. Of eleven MPTR/ETR loci, only five (ETR-A–E) are routinely used for genotyping of *M. tuberculosis* strains. The principle of any typing system based on the polymorphism of VNTR loci is that each VNTR locus is PCR-amplified with specific primers complementary to the flanking regions, and the resulting PCR products are visualized by standard gel electrophoresis (Figure 1(3)). The number of tandem repeat units is determined by estimating the size of the amplicons, in relation to the known size of the repeat unit within the targeted VNTR locus. The results are expressed in a simple, digital format, in which each digit represents the number of copies at a particular locus [63]. Despite being fast, easy-to-perform, and highly reproducible, VNTR genotyping based on 5 ETR loci has a low discriminatory power, compared to IS*6110*-RFLP or spoligotyping [53, 70, 119, 120]. However, with the completion of the *M. tuberculosis* H_37_Rv genome sequencing project, new VNTR-type loci have been identified. A specific class of these new VNTR elements is mycobacterial interspersed repetitive units (MIRUs). MIRUs were originally described by Supply et al. [121] as 46–101 bp tandem repeats scattered at 41 loci throughout the chromosome of *M. tuberculosis* H_37_Rv. Based on the sequence analysis of each of those loci, 12 were found to display variations in tandem repeat copy numbers and were thus selected for genotyping of *M. tuberculosis* isolates [122, 123]. Among those 12 hypervariable loci, two (MIRU-4 and MIRU-31) are identical to formerly described ETR loci (i.e., ETR-D and ETR-E, resp.). MIRU-VNTR analysis, as every VNTR-based typing approach, involves PCR amplification of a specific MIRU locus, followed by determination of the sizes of the amplicons by gel electrophoresis or, after running multiplex PCRs, on an automated, fluorescence-based sequencer (Figure 1(3)). Since the length of the repeat units is known, the calculated sizes reflect the numbers of the amplified MIRU copies. The final result is a multidigit numerical code (the so-called MIRU-VNTR code), corresponding to the repeat number at each analyzed locus [64, 124]. This coding system allows the results to be readily compared across laboratories worldwide and facilitates the data to be deposited in the global databases via the Internet for large-scale epidemiological and population genetic studies [125, 126] (http://www.miru-vntrplus.org/). Recently, the biggest publicly available international database named SITVITWEB, which incorporates multimarker genotyping information (i.e., based on MIRU-VNTR typing and spoligotyping) on 62,582 *M. tuberculosis* complex clinical isolates from 153 countries of patient origin, has been released (http://www.pasteur-guadeloupe.fr:8081/SITVIT_ONLINE/) [104]. Furthermore, a 12-locus MIRU scheme, based on the minimum spanning tress (MST) method, has been proposed for classification of *M. tuberculosis* complex genotypic lineages [127].

The MIRU-VNTR method is a reliable and efficient typing system, whose discriminatory capacity approximates or even exceeds that of IS*6110*-RFLP profiling. In general, the discriminatory power of MIRU-VNTR analysis increases with the number of loci evaluated. MIRU-VNTR typing based on 12 loci is slightly less discriminatory than IS*6110*-RFLP analysis for *M. tuberculosis* isolates with high copy number of IS*6110* [64, 123, 128, 129] but at the same time more discriminatory than the IS*6110*-RFLP if low-copy-number IS*6110* isolates are investigated [130, 131]. In principle, 12-locus MIRU-VNTR cannot be used as a sole typing method, as it may overestimate the number of true epidemiological links, especially in large, population-based studies [132, 133]. Consequently, it is suggested to use 12-locus MIRU-VNTR analysis in combination with other genotyping methods [128, 132]. An alternative way is to increase its resolution by expanding the investigation on other polymorphic VNTR loci.

The observed heterogeneity of the VNTR domains (they are still being discovered in the tubercle bacilli genome) provides great flexibility in designing new marker combinations that would enhance the discriminatory capacity of the genotyping method. In 2006, a new system employing 24 MIRU-VNTR loci (including 12 previously investigated) has been proposed [124]. Noteworthy, 15 (including 6 previously investigated) of those 24 loci account for 96% of all detected polymorphisms in *M. tuberculosis* strains. The discriminatory power of this new, 24-locus MIRU-VNTR typing system equals that of IS*6110*-RFLP profiling [125, 134, 135]. This has rendered 24-locus MIRU-VNTR typing the new gold standard in molecular typing of *M. tuberculosis* complex bacteria. Next to the proposed standardized MIRU-VNTR 15- or 24-loci sets, the use of another three loci, the so-called hypervariable loci (i.e., VNTRs 3232, 3820, and 4120), is recommended as a second-line typing step, particularly to differentiate Beijing genotype strains.

Overall, genotyping based on minisatellite sequences is a rapid, sensitive, and highly discriminating approach, which makes it well suited for large-scale investigations. A particular advantage of the VNTR genotyping, compared to the IS*6110*-RFLP typing, is its portability due to digitalization of the generated patterns and therefore simple intra- and interlaboratory comparability as well as the amenability to inclusion in web-based databases. Reproducibility of the method was expected to be good, due to the genetic stability of the targeted loci; yet, in the first worldwide proficiency study, both intra- and interlaboratory reproducibility proved to be suboptimal. Further harmonization of the laboratory methodology is still needed [136].

The occurrence of VNTR loci in the genomes of nontuberculous mycobacteria remains largely obscure. Eight MIRU-VNTR-type loci have recently been described by Thibault et al. in *Mycobacterium avium* and *M. avium* subsp. *paratuberculosis* [137]. In Japan, a refined VNTR typing method using the *M. avium* tandem repeat (MATR) loci (MATR-VNTR) has been employed [138]. In *M. intracellulare* Dauchy et al. identified 45 potential MIRU-VNTR loci, 7 of which showed enough variability to be used as strain-level discriminatory markers [139]. The discriminatory power of the VNTR typing assays, based on the newly discovered loci in both species, was considered promising [80, 137, 139]. Recently, 13 VNTR loci have been described and applied to confirm clonal relationships between patient and environmental isolates of *M. ulcerans* [140]. Twelve VNTR loci have been defined and tested against *M. marinum* isolates [141].

## 9. Methods Based on GC-Rich Sequences

The genome of *M. tuberculosis* has a particularly high GC content (>65.5%). The polymorphic GC-rich repetitive sequences (PGRS) are the most abundant type of repetitive DNA in the genome of *M. tuberculosis* complex. The PGRS elements occur at multiple loci and consist of several repeats of a 9 bp consensus sequence (5′-CGGCGGCAA-3′), tandemly arranged in up to 1.5 kb segments. Although the PGRS were initially identified in *M. tuberculosis* complex, they are now known to be present in other mycobacterial species, such as *M. kansasii*, *M. gastri*, and *M. szulgai* [142]. The PGRS bear important resemblance to the aforedescribed MPTRs. The similarities between those repetitive elements include host range, structure, genetic stability, and copy number across the mycobacterial genome [143]. Furthermore, PGRS, like MPTR sequences, are part of protein-coding regions. Multiple tandem repetitions of the PGRS-encoded motif AsnGlyGlyAlaGlyGlyAla are found in glycine-rich proteins with a characteristic proline-glutamate (PE) residue group at the N terminus of the peptide. Members of the PE-PGRS family of proteins, analogously to PPE-MPTR protein family, are suspected to play a role in antigenic variability [118].

Since the number of the PGRS element and its distribution vary in different strains, it has been applied as a genetic marker for typing of *M. tuberculosis*. The most extensively used method utilizing this marker is PGRS-RFLP, the procedure of which is quite the same as that for the IS*6110*-RFLP, except that the chromosomal DNA is cut with *Alu*I restriction endonuclease, instead of *Pvu*II, and that a 3.4 kb fragment of the PGRS sequence, cloned in a recombinant plasmid pTBN12, is used as a probe in a hybridization step [142]. The PGRS-RFLP analysis or pTBN12-RFLP fingerprinting has been shown to have a relatively high discriminatory power, especially for IS*6110* low-copy-number strains [56, 144, 145]. The pTBN12 fingerprinting method has similar limitations as the IS*6110*-RFLP typing, but additionally the hybridization patterns produced by PGRS typing are more complex and difficult in interpretation. A second method which benefits from the polymorphism of the PGRS sequence is double-repetitive-element PCR (DRE-PCR). This method relies upon amplification of the DNA segments located between IS*6110* sequences and the PGRS sequences, by using primers directed outwards from the ends of both these repetitive elements (Figure 1(4)). Based on the distance between IS*6110* and PGRS sequences and the copy number of these elements which differs between the strains, DRE-PCR yields strain-specific amplification patterns [65]. Although highly discriminative, the method suffers from poor reproducibility and a strong bias in the interpretation of the results [65, 146, 147].

A similar approach as that reported for DRE-PCR was used to design another typing method, called IS*6110*-ampliprinting [66]. In brief, the method measures the variability in the distances between IS*6110* elements and copies of MPTR sequences of *M. tuberculosis*, through unilateral-nested PCR with IS*6110* and MPTR targeted primers, followed by hybridization with an IS*6110*-specific probe. The method, however, has not been widely adopted, mainly due to the limited number and size of generated PCR products [70].

A recent method that harbors GC-rich sequences, the IS*6110*-Mtb1-Mtb2 PCR typing, is guided by a similar principle as that involved in DRE-PCR profiling. Two PCR assays are performed with primers of two kinds, that is, primers complementary to terminal inverted repeats (TIR) flanking the IS*6110* sequences and primers complementary to short (16 bp) GC-rich motifs, called Mtb1 (I PCR) and Mtb2 (II PCR). As a result, fragments of DNA located between Mtb1 and Mtb2 as well as between each of those elements and IS*6110* sequences are amplified [67]. Although the IS*6110*-Mtb1-Mtb2 PCR method has not been exploited sufficiently, the results from the insofar performed studies suggest its usefulness in the molecular epidemiological investigations of TB. Most notably the discriminatory power of this method has been demonstrated as almost equivalent to that of IS*6110*-RFLP typing [67, 148, 149].

Although PGRS is not commonly being applied for strain typing of NTM, RFLP analysis with PGRS isolated from *M. tuberculosis* as a probe was successfully used to differentiate isolates of the species of *M. kansasii* [150] and *M. ulcerans* [151].

## 10. Repetitive Sequence-Based- (rep-) PCR

Every genotyping method, whose principle is based on the PCR amplification of DNA sequences between repetitive DNA elements, is referred to as repetitive sequence-based- (rep-) PCR. By using primers designed as extended away from the repetitive sequence elements, multiple amplified fragments are generated, depending on the sequence length between the repetitive elements. The amplified fragments produce a fingerprint pattern upon gel electrophoresis. IS*6110* amplityping, DRE-PCR, IS*6110*-ampliprinting, and IS*6110*-Mtb1-Mtb2 PCR, all these methods are representatives of rep-PCR technology. A long list of various repetitive elements that have been applied for genotyping of *M. tuberculosis* and nontuberculous mycobacteria includes also DNA sequences that lack species or even genus specificity, such as enterobacterial repetitive intergenic consensus (ERIC) and the (GTG)_5_ sequences [152–155].

Recently, a commercially available rep-PCR system (DiversiLab System, bioMérieux, France) has been adapted for use on mycobacteria. The DiversiLab System, which takes advantage of various repetitive elements interspersed throughout different bacterial genomes, was evaluated on a collection of *M. tuberculosis* and *M. avium* complex isolates [156, 157] as well as *M. abscessus* isolates [158]. For *M. tuberculosis* and *M. avium* subsp. *avium*, the discriminatory ability of the assay equaled or exceeded that of IS*6110*-RFLP and IS*1245*-RFLP [156, 157, 159]. In more recent reports, rep-PCR was successfully used to disclose the source of infection in a patient with hypersensitivity pneumonitis caused by *M. avium* [160].

## 11. Random Amplified Polymorphic DNA (RAPD) Analysis

Random amplified polymorphic DNA (RAPD) analysis, also referred to as arbitrary primer PCR, depends on amplification of random fragments of genomic DNA using arbitrarily designed primers (5 to 50 bp) under low stringency conditions. The interstrain polymorphism is assessed by comparing the electrophoretic pattern of the PCR products. Although the method provides high discriminatory power, a number of limitations exist for this technique, of which the apparent lack of reproducibility is the most important. This is because differences between the strain patterns generated by RAPD are due to technical and operating parameters of the method rather than true interstrain genetic polymorphism. Variations in RAPD patterns are chiefly attributed to variations in the priming efficiency during early rounds of amplification, and these in turn depend on template concentration and purity, primer/template ratio, or the ramp times of the cyclers used. Nonetheless, the RAPD typing has been used to differentiate among isolates of *M. tuberculosis* complex isolates [161–164] as well as among isolates of numerous NTM species, including *M. abscessus* [165], *M. phocaicum* [166], *M. gordonae* [167], *M. szulgai* [168], and *M. malmoense* [169].

The RAPD analysis is usually performed on the entire genomic DNA; however in some modifications of the method only a fragment of the bacterial chromosome is used as a substrate for PCR. Such a strategy was employed for genotyping of *M. tuberculosis* strains by Abed et al., who used the 16–23S rDNA internal transcribed spacer (ITS) region within the rDNA operon as a target for PCR. The subsequent RAPD analysis of the amplified product resulted in highly polymorphic and easily interpretable profiles [170]. Still, the reproducibility of this RAPD-based method was found to be poor [171, 172].

## 12. Amplified Fragment Length Polymorphism (AFLP) Analysis

Amplified fragment length polymorphism (AFLP) analysis is a PCR-based method in which genomic DNA undergoes digestion with two restriction enzymes, a rare cutter and a frequent cutter, typically having six- and four-nucleotide-long recognitions sites, respectively (e.g., *Eco*RI and *Mse*I, resp.), followed by ligation of two types of synthetic, double-stranded adaptors (10–30 bp) to the generated cohesive ends of DNA fragments. Amplification of a subset of restriction fragments is achieved by PCR with primers complementary to the adaptor sequence, the restriction site sequence, and a few nucleotides (1–3) extending beyond the restriction site. The amplicons are separated and visualized on denaturing polyacrylamide gels, usually through autoradiography, as the primers are radioactively labeled [173]. The conventional radioactive AFLP method has a lower discriminatory potential than IS*6110*-RFLP typing [174, 175]. However, the AFLP technology provides an extraordinary flexibility in designing the typing protocols of enhanced power of discrimination. Here, the choice of restriction endonucleases and the degree of primer selectivity may largely determine the final resolution of the method.

A refinement of the traditional AFLP assay is the fluorescent (f)AFLP, where the subsets of the DNA digestion fragments are PCR-amplified with five primers, a single, nonselective, unlabeled forward primer targeting the *Mse*I adaptor site and four reverse primers, targeting the *EcoR*I adaptor site, each containing the selective base A, G, C, or T, labeled with different fluorescent dyes. The amplified fragments are resolved by electrophoresis on an automated DNA sequencer and precisely sized using internal size standards [176]. Apart from the improved occupational safety, the discriminatory potential of fAFLP is higher than that of the radioactive AFLP and comparable to that achieved by IS*6110*-RFLP [176, 177].

The AFLP method has successfully been applied to various NTM species including the *M. avium* complex [178], *M. haemophilum* [179], and *M. marinum* and *M. ulcerans* [180], although its exact discriminatory power in larger sets of isolates has never been assessed.

Furthermore, AFLP analysis has clearly distinguished between *M. marinum* and *M. ulcerans*, conforming that these species which are difficult to distinguish by conventional methods, are genetically distinct [180, 181].

## 13. Multilocus Sequence Typing (MLST)

Genetic polymorphism between and within different mycobacterial species can also be investigated at the nucleotide sequence level. Such a concept gave rise to a new typing system, the so-called multilocus sequence typing (MLST), which evolved directly from the multilocus enzyme electrophoresis (MLEE or MEE) technique. Unlike MLEE, which targets the electrophoretic mobility of slowly evolving metabolic enzymes (usually 15 to 25), isolated from each bacterial isolate, MLST targets the sequences of a small number (up to 10) of housekeeping genes coding for vital enzymes and structural proteins. Although both MLEE and MLST schemes failed to reveal sufficient polymorphism among members of the *M. tuberculosis* complex [27, 182], these techniques were found useful for differentiation of various NTM species. For instance, by using MLEE, polymorphism was found among isolates of the *M. avium-M. intracellulare* complex [183–185]. On the other hand, the MLST analysis, based on 10 genetic loci, allowed the determination of the variability between subspecies and strains of *M. avium* and thus greatly improved our knowledge on genetic divergence and evolution of this group of NTM [186]. MLST has also proven to be valuable in investigations of NTM in laboratory outbreak settings [166].

Given the low degree of sequence polymorphisms in *M. tuberculosis* complex, standard MLST is poorly informative and inefficient. Its place has now been taken over by a new typing strategy based on analysis of single nucleotide polymorphisms (SNPs).

## 14. Single Nucleotide Polymorphism (SNP) Typing

Single nucleotide polymorphisms (SNPs) fall into two major groups: synonymous- (s-) SNPs and nonsynonymous- (ns-) SNPs. The latter, if present in coding regions, introduce amino acid changes to the proteins. This in turn may influence the phenotype and be subjected to selection pressure. For instance, ns-SNPs are implicated in *M. tuberculosis* resistance to anti-TB drugs (drug resistance in *M. tuberculosis* is almost invariably associated with mutations (nonsynonymous point mutations, small deletions, and duplications) in specific, chromosomal loci) [187]. Screening for ns-SNPs in resistance-conferring genes provides important insights into the molecular mechanisms and dynamics of the development of drug resistance.

Contrariwise, s-SNPs do not alter the amino acid profiles and are thus phenotypically neutral. As s-SNPs are also believed to be evolutionary neutral, they are used for population genetics and for studying phylogenetic relationships among mycobacterial strains [188, 189]. A picture of the phylogenetic population structure of *M. tuberculosis* has recently been inferred by using a combination of s-SNP and non-SNP markers. Based on the SNPs at codon 463 of the *katG* gene and codon 95 of the *gyrA* gene, Sreevatsan et al. divided *M. tuberculosis* complex into 3 principal genetic groups (PGG1–PGG3) [14]. Those three PGG groups were further split into 9 major clusters (I–VIII and II.A) by analysis of additional 36 s-SNPs [189]. Recently, Dos Vultos et al. [190] have indicated SNPs in the 56 genes encoding 3R (DNA replication, recombination, and repair) system components as the key genetic markers to study the evolution of *M. tuberculosis*.

Although SNPs represent the most reliable markers for lineage classification of MTBC, their use is hampered by the need to test a large set of genes to achieve satisfactory resolution. Recently, Homolka et al. [16] have developed a SNP-based diagnostic algorithm allowing the identification of 17 MTBC phylogenetic lineages with high specificity. The algorithm involves sequence analysis of only five genes. The SNP typing approach is highly specific and sensitive, although SNP analysis has predominantly been used in genealogy, phylogenetic, and population genetics studies.

## 15. Deletion Mapping and Deligotyping

Comparative genomic studies of different strains of *M. tuberculosis* (e.g., H_37_Rv, CDC1551) have proven that the loss of genetic material has stigmatized the evolutionary history of that species. Genomic deletions, also known as large-sequence polymorphisms (LSPs) or regions of difference (RD), have been detected across the *M. tuberculosis* genomes [191, 192]. For example, a total of 68 distinct deletions, ranging in size from 105 bp to approximately 12 kb, were found in 100 *M. tuberculosis* clinical isolates [192]. Deletions are not randomly distributed but rather appear in aggregations. They occur within both intra- and intergenic regions [191–194]. Noteworthy, almost half of the LSPs identified in *M. tuberculosis* H_37_Rv and CDC1551 strains involved genes encoding PPE and PE family proteins [191]. Since LPSs have emerged as a significant source of interstrain genetic variability, they have been used as markers for genotyping. Analysis of chromosomal deletions has been shown as an extremely attractive approach for studying the phylogeny and evolution of *M. tuberculosis* complex [192, 195, 196]. Deletion analysis, also referred to as deligotyping, can be performed either by a simple PCR-based method or by automated microarray techniques. The resolution of the method can greatly be increased if specific sequences flanking each side of the deletion element are known. Recently, a high-throughput method for distinguishing LSPs was invented. Both the concept and procedure of this method were patterned upon the spoligotyping technique. Here, deletion events are detected in 43 genomic loci by amplifying them in a multiplex PCR assay and subjecting the amplicons to hybridization with a set of 43 probes, whose sequences directly correspond to the targeted loci [197]. Deligotyping is a very sensitive and efficacious approach for rapid screening of clinical isolates of *M. tuberculosis*. The method is also well suited for constructing robust phylogenetic relationships [198].

## 16. Concluding Remarks

As described above, there is a wide range of methods available for genotyping of *M. tuberculosis* complex and NTM species (Tables 1 and 2). Each method has its own benefits and shortfalls, and none of them have proven clearly superior to any of the others. The choice of the optimal typing system depends heavily on the sample under investigation, the setting in which typing is performed, and the expected outcome. For instance, spoligotyping is of particular value in population-based studies to define the phylogeographic specificity of circulating clades of tubercle bacilli. However, to assess the genetic relatedness and the epidemiological links among TB outbreak-related cases, the IS*6110*-RFLP or MIRU-VNTR typing is preferably chosen. Again, whereas spoligotyping is recommended as a preliminary screening test of a large number of *M. tuberculosis* isolates, disclosure of true genetic relationships between the isolates requires more discriminating methods, such as MIRU-VNTR typing, to be performed within the spoligotype-defined clusters.

An ideal molecular typing method should accommodate the requirements with respect to both performance and analytical criteria. The desired performance parameters include technical simplicity (easiness of performance), reproducibility, robustness, and time and cost effectiveness. An attractive feature of the method could be its applicability directly to clinical material. Another special advantage is a standardized and easily portable and interpretable format of the results (e.g., digital codes), facilitating databasing and interlaboratory comparative studies. As for the analytical parameters, the most important are the level of discrimination and stability of the genetic marker used. The general rule that the higher the discriminatory power of a given method, the more reliable the results obtained guides most of the molecular epidemiology investigations. However this may not always be the case. The validity of such assumption depends on several issues related to clustering, such as the characteristics of the study setting, the proportion of cases included (completeness of sampling), or the period of case recruitment (duration of the study). In other words, a number of important considerations have to be taken into account when choosing an appropriate typing methodology in terms of discrimination capacity.

The discriminatory power of molecular markers is directly linked to the genetic stability of each marker. There have been observed minor changes in DNA fingerprint patterns of *M. tuberculosis* strains isolated not only from epidemiologically related TB cases, but also from the same patient at different points of time [52, 199–202]. The stability of the genotypic patterns over time reflects the evolutionary rate, also referred to as a “molecular clock,” of each genetic marker. For example, a half-life of the IS*6110*-RFLP profiles has been shown to be much shorter than that of the spoligotype profiles [70, 98]. Likewise, the combined molecular clock of the MIRU-VNTR loci has been shown to be slower than that of IS*6110*-RFLP [203]. In general, the best molecular marker is the one, whose “molecular clock” is, on the one hand, fast enough to distinguish unrelated cases and, on the other hand, sufficiently slow to capture epidemiologically linked cases [204]. Markers evolving rapidly and those evolving at slow rates may either underestimate or overestimate the amount of recent transmission of the disease, respectively.

The choice of a genotyping method, with respect to its discriminatory ability, depends on the type of research in question. Whereas a highly discriminatory method, that is with a fast “molecular clock,” would be required to determine whether an infection is a reactivation of an infection acquired in the past (latent infection) or rather a reinfection with a new strain, a method with a slow “molecular clock” would be needed for global strain tracking and evolutionary studies.

The resolution power of different typing methods, yielding very diversified genetic patterns, has had an impact on the definition of clustering. Indeed, there persists a controversy in the literature on whether or not isolates whose patterns show subtle differences, that is, of 1-2 bands, in the IS*6110*-RFLP patterns or single locus variations (SLVs) in the MIRU-VNTR patterns may be regarded as part of a genetic cluster and thus constitute an ongoing chain of transmission. Whereas some authors apply a strict cluster definition including only isolates with identical genotypes [134, 135, 205, 206], the others advocate the use of slightly relaxed criteria for defining clusters, with a tolerance of a single- or double-band difference in the RFLP profiles and/or SLVs or double locus variations (DLVs) in the MIRU-VNTR profiles [52, 200, 207, 208]. With the latter approach, another important question arises, that is, to what extent the fingerprint patterns of two isolates may differ before they are no longer considered to be clustered. Since no clear cut-offs exist for defining a cluster, the decision which isolates should be included or excluded from clusters is largely a matter of arbitrariness. In general, the more lenient the assumed criteria, the higher the chance of detecting clusters, but the lower the likelihood that a cluster represents epidemiologically related cases [209].

One has also to be mindful that the identity or high similarity of DNA fingerprints from two individuals, obtained by using even the most discriminating techniques, may not always mean a recently transmitted infection. Other explanations are possible, including the simultaneous reactivation of a previously acquired infection with the same organism (coincidence of time), the regional predominance of a particular strain, circulating over a long time, or a laboratory cross-contamination [210].

Identical genotypic patterns may not indicate clonality, even when multiple genetic markers are employed. This was best evidenced by Niemann et al. who compared the complete genomes of two *M. tuberculosis* Beijing genotype isolates from a high-incidence region (Karakalpakstan, Uzbekistan), of which one was drug susceptible and the other was multidrug resistant. Both isolates shared the same IS*6110*-RFLP pattern and the same allele at 23 out of 24 MIRU-VNTR loci, yet they differed by 130 SNPs and one large deletion [211]. This finding implies some important messages. First, *M. tuberculosis* isolates exhibiting identical DNA fingerprinting profiles may still display substantial genomic diversity. This in turn may lead to misinterpretation of the extent of TB transmission in a community or invalid differentiation between disease relapse and exogenous reinfection, when using standard genotyping tools. It seems that the optimal option to fully explore the phylogenetic branching and variation on strain level and to justifiably draw epidemiological conclusions on the etiology, host-range, and transmission of TB disease would be the application of whole-genome sequencing (WGS) analysis. Although still an expensive solution, with the plummeting cost of DNA sequencing, the WGS may become an omnipotent approach to replace all previously known diagnostic tests for *M. tuberculosis*, including those for identification or drug susceptibility profiling [212]. As for the present, a status of the “gold standard” typing method for *M. tuberculosis* is still being held by the IS*6110*-RFLP. However, it is being increasingly replaced by the MIRU-VNTR typing, not only because its discriminatory power equals that of IS*6110*-RFLP but only because it has methodological and practical advantages over RFLP, most important of which is that the PCR-based typing method requires fewer bacteria and consequently shortens considerably delay in obtaining genotypes. Thus, the MIRU-VNTR typing can be regarded as the new standard for TB molecular epidemiology.

**Figure 2:**
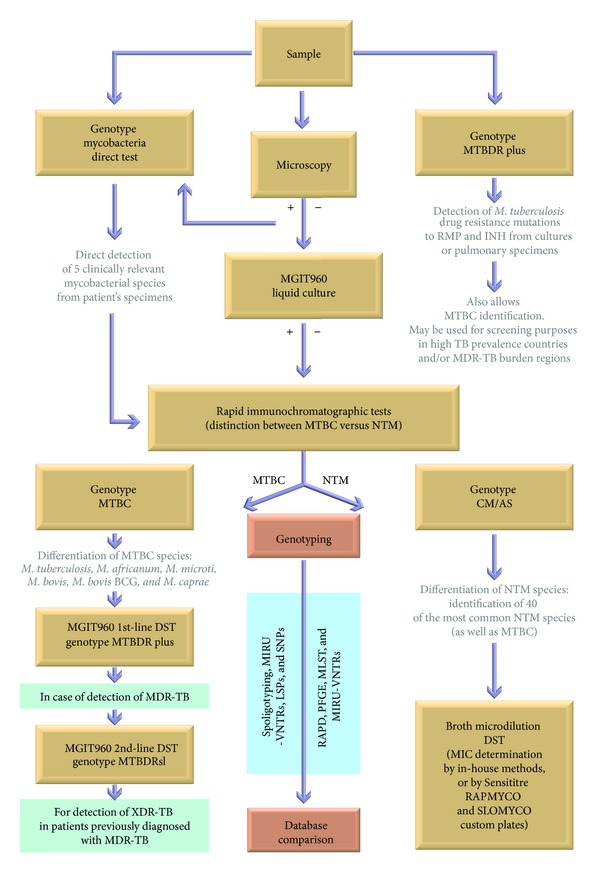
Schematic flow diagram illustrating processing of clinical samples for species identification, drug susceptibility testing (DST), and genotyping of mycobacteria belonging to *Mycobacterium tuberculosis* complex (MTBC) and nontuberculous mycobacteria (NTM). MIRU-VNTRs: mycobacterial interspersed repetitive units-variable number of tandem repeat loci; LSPs: large sequence polymorphisms; SNPs: single nucleotide polymorphisms; RAPD: random amplified polymorphic DNA; PFGE: pulsed-field gel electrophoresis; MLST: multilocus sequence typing.

As for the NTM, a number of genotyping methods are available, most of which were first applied to *M. tuberculosis* complex and then turned out to work for specific NTM species, but the discriminatory power of those methods has not been fully examined. A method most commonly applied in various NTM species, with only slight species-specific modifications, has been PFGE typing. Due to its wide use, it could be considered the “gold standard” for all NTM species except for *M. avium*. For the latter, IS*1245* RFLP typing is the widely recognized reference method [79]. MLST has also the potential to become a reference method, although its wider use is hampered by limited access and high costs of the sequencing facilities. However, with the level of genetic diversity in NTM (sub)species being ill-defined and the discriminatory power of most of the markers not fully established, for a proposal of the “gold standard” typing system for NTM species to be delivered, further studies are required (Figure 2).

Although there is currently no genotyping method that would work in diverse settings and population groups or to be equally effective at answering particular epidemiological questions, the application of molecular typing methods has significantly advanced our knowledge of the transmission and pathogenesis of mycobacteria.

Molecular typing methods have permitted investigation of the outbreaks [36, 61, 100, 139, 165, 166, 213, 214], discrimination between exogenous reinfection and endogenous reactivation [209, 210, 215–217], identification of mixed infections [215, 218, 219], and cases of misdiagnosis due to laboratory contamination [220–223]. Molecular markers have also extensively been used for tracking transmission patterns within specific populations and/or defined geographical settings [106–108, 153, 206, 224–227]. Finally, genotyping methods have shed important light on the phylogeny and evolutionary history of the mycobacterial species [12, 14, 189, 194].

Methods of molecular typing constitute an integral element of virtually all epidemiological studies on mycobacterial infections. They continue to substantially improve our understanding of the biology of mycobacteria and are believed to provide novel and powerful tools to combat and/or protect against the diseases caused by these pathogens.

## Figures and Tables

**Table 1:** Selected typing methods for *Mycobacterium tuberculosis* complex and level of genetic polymorphism they reveal.

Typing method	DNA target	Polymorphism	References*
IS*6110*-RFLP	IS*6110 *	High	van Embden et al., 1993 [48]
ML-PCR		High	Haas et al., 1993 [58]
FliP		High	Reisig et al., 2005 [59]
LM-PCR		High	Prod'hom et al., 1997 [60]
HIP		High	Kearns et al., 2000 [61]
Spoligotyping	DR locus	Low	Kamerbeek et al., 1997 [62]
VNTR typing	ETRs A–E	Low	Frothingham and Meeker-O'Connell, 1998 [63]
MIRU-VNTR typing	MIRUs	High	Supply et al., 2001 [64]
DRE-PCR	IS*6110*/PGRS	High	Friedman et al., 1995 [65]
IS*6110*-ampliprinting	IS*6110*/MPTR	High	Plikaytis et al., 1993 [66]
IS*6110*-Mtb1-Mtb2 PCR	IS*6110*/Mtb1/Mtb2	High	Kotlowski et al., 2004 [67]

*Papers with original description of a given method.

RFLP: restriction fragment length polymorphism; ML-PCR: mixed linker PCR; FliP: fast ligation-mediated PCR; LM-PCR: ligation-mediated PCR; HIP: heminested inverse PCR; VNTRs: variable numbers of tandem repeats; MIRU: mycobacterial interspersed repetitive units; DRE-PCR: double-repetitive-element PCR; DR: direct repeat; ETR: exact tandem repeat; PGRS: polymorphic GC-rich sequence; MPTR: major polymorphic tandem repeat.

**Table 2:** Discriminatory power of selected typing methods for *Mycobacterium tuberculosis* complex and nontuberculous mycobacteria and level of genetic polymorphism they reveal.

Typing method	MTBC	NTM
RFLP	High	Insufficient data
RAPD	Medium	High
PFGE	Medium	High
AFLP	High	Insufficient data
Spoligotyping	Low/diverse	None
MIRU-VNTR typing	High/diverse	Insufficient data

MTBC: *Mycobacterium tuberculosis* complex; NTM: nontuberculous mycobacteria; RFLP: restriction fragment length polymorphism; RAPD: random amplified polymorphic DNA; PFGE: pulsed-field gel electrophoresis; AFLP: amplified fragment length polymorphism; MIRU-VNTR: mycobacterial interspersed repetitive unit-variable number of tandem repeats.
